# Supplementary material for: Nanofiber Space-Confined Fabrication of High-Performance Perovskite Films for Flexible Conversion of Fluorescence Quantum Yields in LED Applications
Source: Polymers (Basel). 2024 Sep 11;16(18):2563. doi: 10.3390/polym16182563 (PMC11434933; doi:10.3390/polym16182563)
Supplement: Supplementary file 1 [file polymers-16-02563-s001.zip › polymers-3148874-supplementary.pdf]

Supporting Information

Nanofiber space-confined fabrication of high-performance perovskite films for flexible fluorescent quantum conversion in LED application

Ningbo Yi[1,2,5], Xue Guan[1], Xiaoting Chen[1], Luojia Xie[1], Nan Zhang[2,3,4], Jinfeng Liao[3], Long Su[1,3], Yancheng Wu[1], Feng Gan[1], Guoqiang Chang[5], Liyong Tian*[,1], Yangfan Zhang*[,1]

[1] College of Textile Science and Engineering, Wuyi University; Jiangmen, P. R. China; yiningbo@wyu.edu.cn (N.Y.); gxykxy611@163.com (G.X.); upting-chen@outlook.com (X.C.); 15625631178@163.com (L.X.); longsu.81102@gmail.com (L.S.); yancheng_wu@126.com (Y.W.); gf@dhu.edu.cn (F.G.)

[2] Guangdong Provincial Key Laboratory of Semiconductor Optoelectronic Materials and Intelligent Photonic Systems, Harbin Institute of Technology, Shenzhen, P. R. China; yiningbo@wyu.edu.cn (N.Y.); nanzhang@xjtu.edu.cn (N.Z.)

[3] Joint Key Laboratory of the Ministry of Education, Institute of Applied Physics and Materials Engineering, University of Macau, Macau, P. R. China; nanzhang@xjtu.edu.cn (N.Z.); jinfengliao@um.edu.mo (J.L.); longsu.81102@gmail.com (L.S.)

[4] MOE Key Laboratory for Nonequilibrium Synthesis and Modulation of Condensed 5 Matter, School of Physics, Xi`an Jiaotong University, Xi'an, P.R. China; nanzhang@xjtu.edu.cn (N.Z.)

[5] Kai Rong De（Shao guan）Fibre Glass Co.,Ltd, Shaoguan, P. R. China; changguoqiang@kingboard.com (G.C.);

* Correspondence: tlydbd@163.com (L.T.); zyf@wyu.edu.cn (Y.Z.)

Supplementary figures

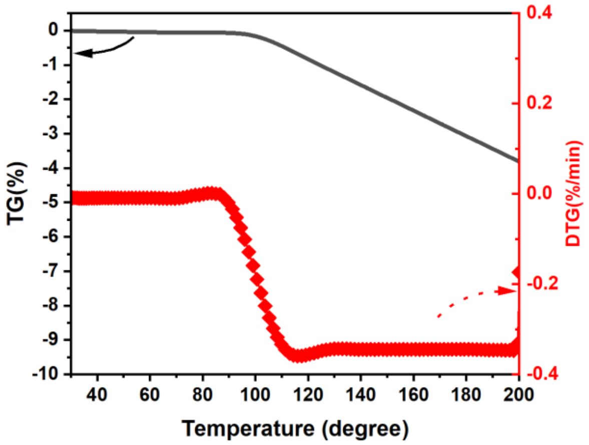

Figure S1. TGA-DTA analysis on perovskite/PAN composite under an O_2 atmosphere.

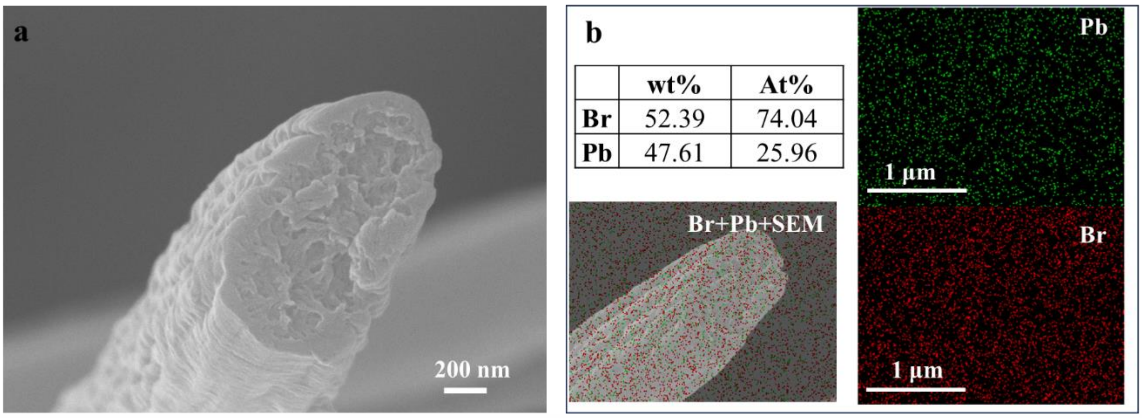

Figure S2. Cross-sectional SEM (a) and elements mapping (b) of composite fiber with high-resolution.

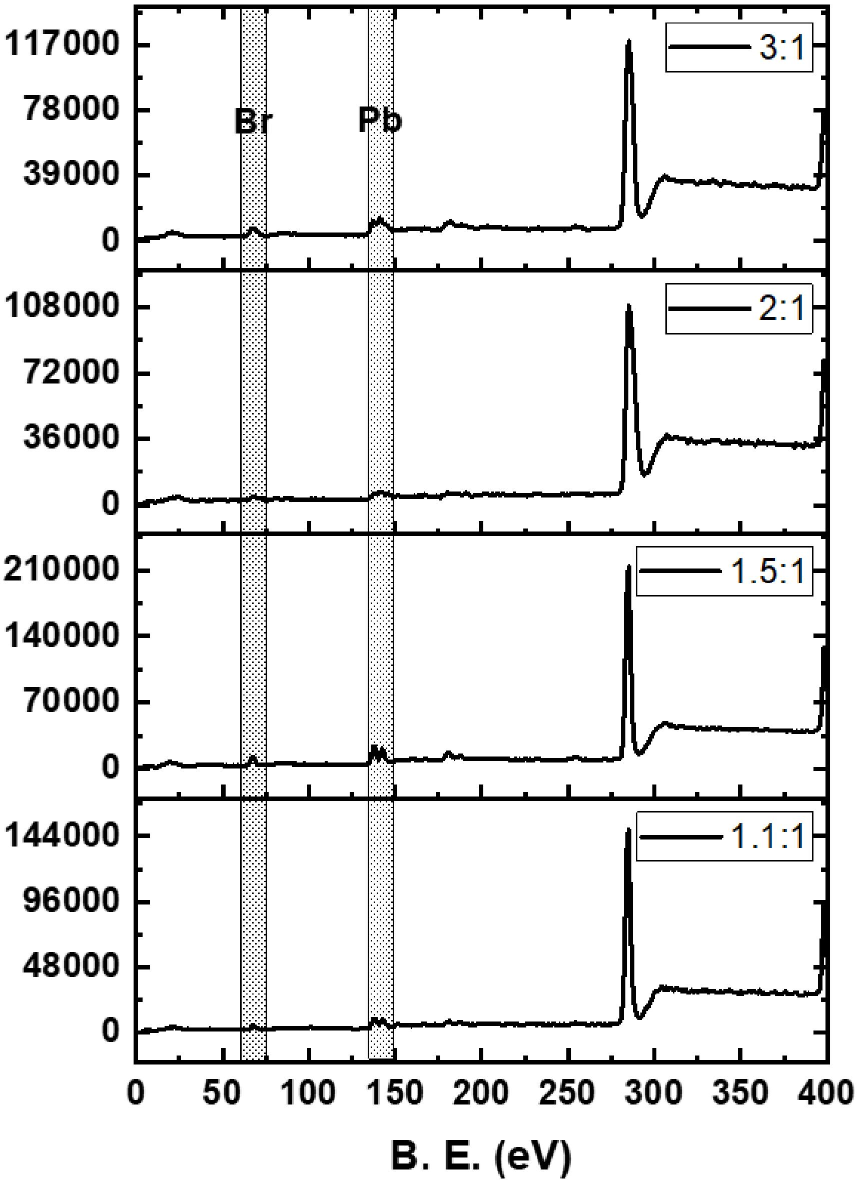

Figure S3. The XPS analysis of QCMs with different ratio of $MABr/PbBr_2$ in the precursor solution for electrospinning.

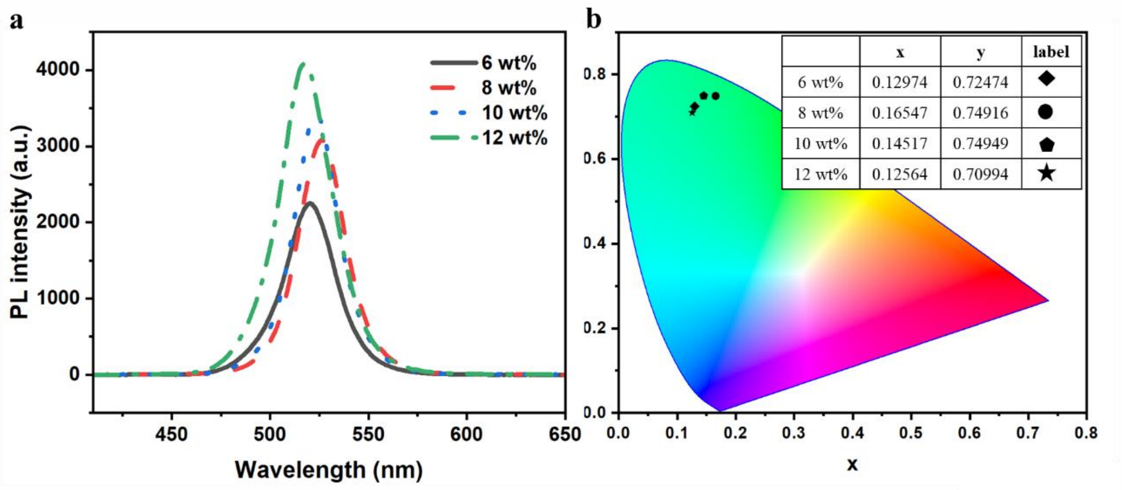

Figure S4. PL spectra and relevant CIE color coordinates of perovskite/PAN composite film with different contents of perovskite.

electrospinning.

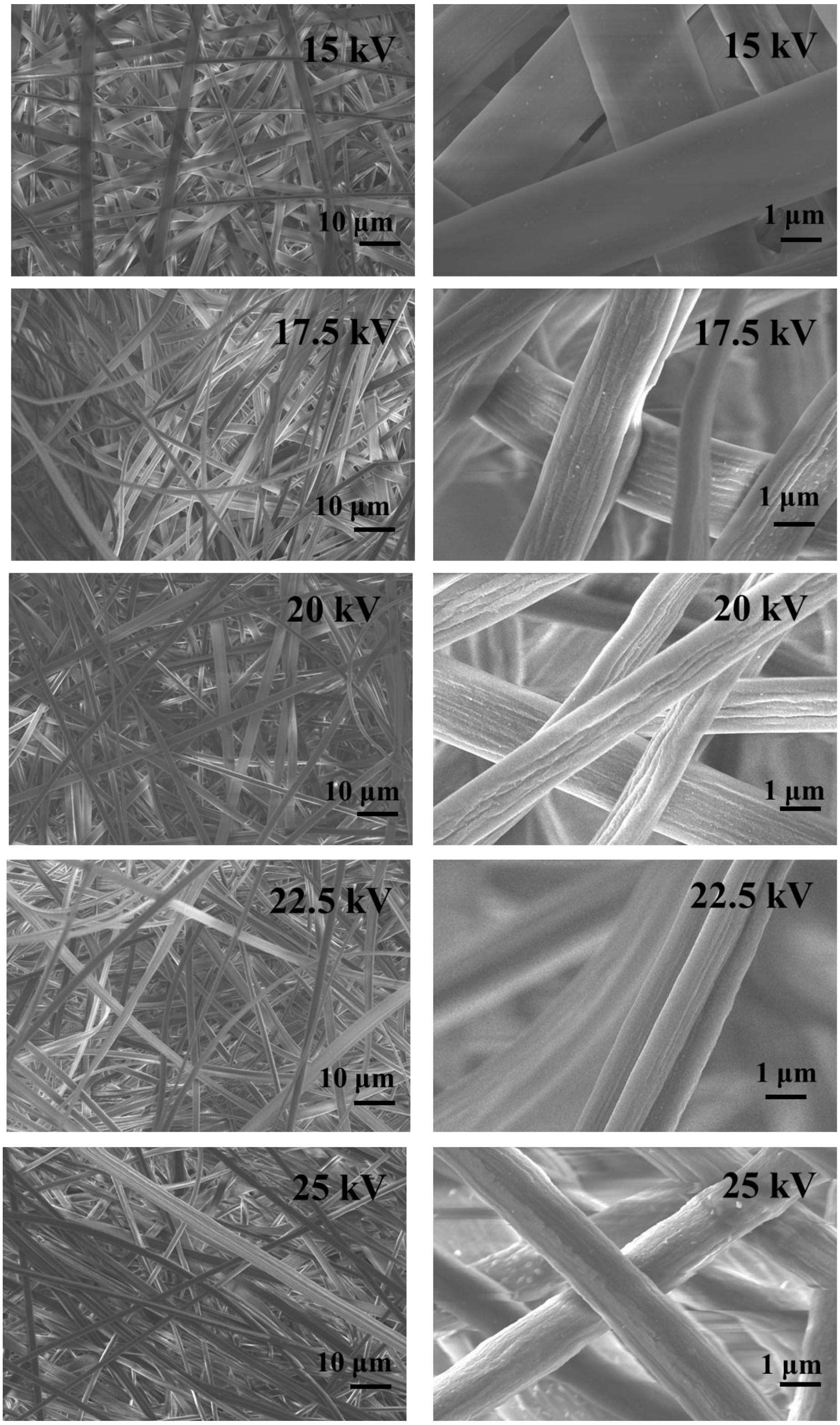

Figure S5. SEM images of QCM structures obtained under different high voltages applied in electrospinning.

Table S1. PLQY of $MAPbBr_3$-based composites with different polyers

perovskite	Polymer matrix	PLQY	Ref.
$MAPbBr_3$	PCL	96.8%	28
$MAPbBr_3$	PVDF	85.9%	29
$MAPbBr_3$	PS-b-PEO	43%	30
$MAPbBr_3$	PS	46%	31
$MAPbBr_3$	PMMA	93.86%	32
$MAPbBr_3$	silicone	62%	33
$MAPbBr_3$	PMMA	60%	34
$MAPbBr_3$	polycarbonate	75%	35
$MAPbBr_3$	Ethyl Cellulose	65.1%%	36
$MAPbBr_3$	PAN	80%	Our work
